# Optimization of cationic polymer-mediated transfection for RNA interference

**DOI:** 10.1590/1678-4685-GMB-2021-0237

**Published:** 2022-03-09

**Authors:** Xiaojie Fan, Jingnan Yang, Guangyao Wu, Meiyi Wang, Xiaoxia Cheng, Chang Liu, Qian Liu, Yanan Wen, Shuangshuang Meng, Zhenxing Wang, Xuhong Lin, Lei An

**Affiliations:** 1 Huaihe Hospital of Henan University, Kaifeng, Henan, China.; 2 Henan University, School of Medicine, Kaifeng, Henan, China.

**Keywords:** shRNA, transfection reagent, cationic polymer, RNAi.

## Abstract

Transfection efficiency was estimated to optimize the conditions for RNA interference (RNAi), including transfection time, validity, and nucleic acid concentration and type, using the EZ Trans Cell Reagent, a cationic polymer. An shRNA against GFP was designed and transfected into cells using the EZ transfection reagent. The shRNA significantly decreased the expression of GFP. In addition, pre-diluted transfection reagent at room temperature and small nucleic acids increased the transfection efficiency, which peaked at 24 h. Compared with circular nucleic acids, linear nucleic acids showed higher transfection efficiency and a higher genome integration rate. We optimized cationic polymer-mediated RNAi conditions, and our data will be useful for future RNAi studies.

## Introduction

RNA interference (RNAi) is a conserved mechanism of gene silencing in a variety of cell types (Han, 2018). RNAi is mediated by double-stranded RNA (dsRNA) and was first observed in the nematode C. elegans (Fire et al., 1998). RNAi is used to investigate the biological effects of loss-of-function mutations in individual genes (Rosa et al., 2018). In addition, RNAi has been used for gene therapy (Bobbin and Rossi, 2016).

Plasmids carrying dsRNA must enter the cell to mediate gene silencing. Multiple methods have been developed to transfer siRNAs into cells, such as cationic lipid/polymer-mediated transfection, electroporation, and virus-mediated transduction (Longo et al., 2013; Ozbalci et al., 2019). Electroporation is rapid and economical but can reduce cell viability (Sherba et al., 2020; Zhang et al., 2018). Virus-mediated transduction is efficient but complex and has a high technical threshold (Zuk, 2011). Cationic lipid/polymer reagents have high transfection efficiency and low cytotoxicity and are suitable for a range of cell types (Montoya and Azorsa, 2016; Kumar et al., 2019). Cationic lipids or polymers are typically used for cell transfection.

EZ trans cell transfection reagent is a new generation of transfection reagent, and its transfection principle is that the positively charged cationic polymer forms a positively charged complex with the negatively charged phosphate group in the nucleic acid, interacts with the negatively charged proteoglycan on the cell surface, and enters the cell through endocytosis.

However, their transfection efficiency is influenced by multiple factors, such as transfection time and plasmid and shRNA/siRNA concentrations (Bollin et al., 2011; Swiech et al., 2011; Perrone et al., 2013). To improve transfection efficiency, it is necessary to optimize the transfection conditions. We optimized the conditions of polymer-mediated RNAi mediated by the EZ Trans Cell Reagent, a cationic polymer.

## Material and Methods

### Reagents and antibodies

Fetal bovine serum (FBS) and Dulbecco’s modified Eagle’s medium (DMEM) were purchased from Gibco (USA). EZ Trans Cell Reagent was purchased from Shanghang Life iLAB Biotechnology Co., Ltd. (Cat:AC04L098, China, http://www.life-ilab.com.cn/Products-35605022.html). Culture medium, serum, and antibiotics were purchased from Gibco and PBS from Procell (USA). DNA ligase was purchased from New England Biolabs (USA). The PMD-18T recombinant plasmid was generated in our laboratory.

### Plasmid construction

The PMD-18T recombinant plasmid encoding green fluorescent protein (GFP)-shRNA was constructed by PCR. The sequence of the GFP-shRNA was GAGGGCCTATTTCCCATGATTCCTTCATATTTGCATATACGATACAAGGCTGTTAGAGAGATAATTAGAATTAATTTGACTGTAAACACAAAGATATTAGTACAAAATACGTGACGTAGAAAGTAATAATTTCTTGGGTAGTTTGCAGTTTTAAAATTATGTTTTAAAATGGACTATCATATGCTTACCGTAACTTGAAAGTATTTCGATTTCTTGGCTTTATATATCTTGTGGAAAGGACGAAACACC GCAAGCTGACCCTGAAGTTCA TTCAAGAGA TGAACTTCAGGGTCAGCTTGC TTTTTT

(Underline, human U6 promoter; green highlight, GFP sequence of shRNA target; blue highlight, spacer sequence [loop]; pink highlight, complementary sequence of shRNA target; red highlight, TTTTTT transcription termination signal.) The linear DNA plasmid was synthesized by Shanghai Sangon Company (China).

### Cell culture and transfection

293T cells were cultured in DMEM supplemented with 1% penicillin/streptomycin and 10% FBS at 37 °C with 5% CO_2_. Cells in the logarithmic phase were harvested and seeded in cell culture dishes. Next, the cells were transfected with DNA plasmid and EZ Trans Cell Reagent at a 1:3 ratio (µg:µL) according to the manufacturer’s instructions. Assays were performed at 12, 24, and 48 h after transfection.

### Immunohistochemistry

Cells resuspended on slide were fixed in solution I (0.1 M PB containing 4% paraformaldehyde) for 1 h at room temperature (RT). After washing three times with 0.1 M PB for 30 min, cultured cells were incubated with the primary antibodies (mouse anti-tubulin and anti-vinculin) in solution II (1% bovine serum albumin in 0.1 M PB containing 0.1% Triton X-100) overnight at 4 **°**C. After washing three times in 0.1 M PB for 30 min, the cells were incubated with the secondary antibody (Alexa Fluor 568-conjugated goat anti-mouse [1:300, 2 mg/mL]; Invitrogen [USA]) in solution III (0.1 M PB containing 0.1% sodium azide) for 2 h at RT. Some cells were stained for F-actin using tetramethylrhodamine-conjugated phalloidin (1:4200, 1 mg/mL; Millipore [USA]). The cells were counterstained with DAPI (1:300, 0.1 mg/mL; Millipore). Fluorescent image analysis algorithms on fluorescent microscope and stained cells were performed under a microscope (Ti2-U; Nikon [Japan]).

### Stability of premixed small nucleic acids

GFP was used as the simulant. The premixed liquid was prepared, and half of the volume was diluted with PBS. Next, one quarter volumes of the diluted and non-diluted solutions were stored at 4 °C, and another quarter volume was stored at RT. After storage, the cells were transfected and analyzed after 14, 24, and 48 h.

### Statistics analysis

Statistical analysis was performed using SPSS 18.0 software. Continuous variables are expressed as means ± standard deviation (SD) and were compared between two groups by Student’s *t*-test. Comparisons among three or more groups were performed by ANOVA. All analyses were two-tailed, and *p* < 0.05 was considered to indicate statistical significance.

## Results

### Optimal transfection time

**Figure 1 -:**
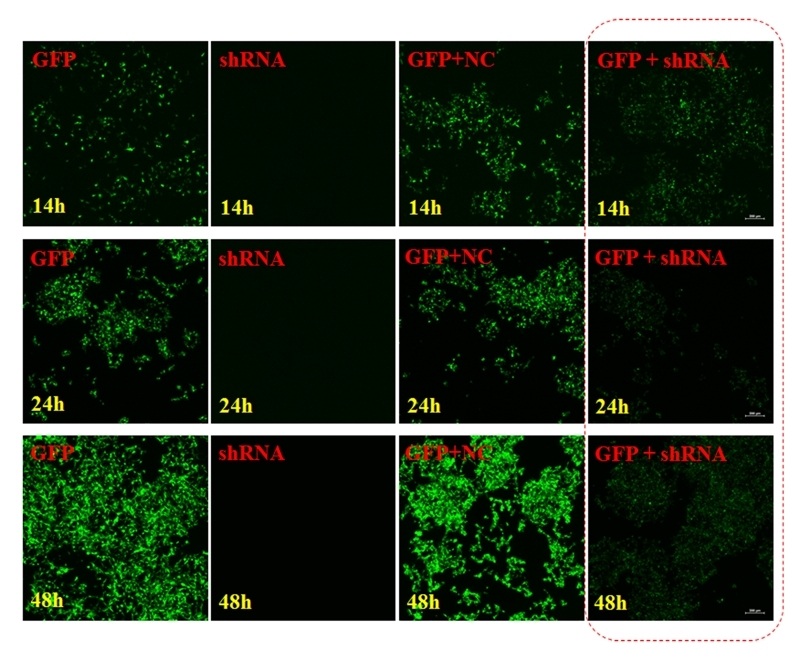
In the GFP, single small nucleic acids, mixed GFP + NC, mixed GFP + small nucleic acids, and shRNA groups, transfection of the PMD-18T recombinant plasmid encoding GFP-shRNA significantly reduced GFP expression at 14, 24, and 48 h, with a nadir at 24 h.

**Figure 2 -:**
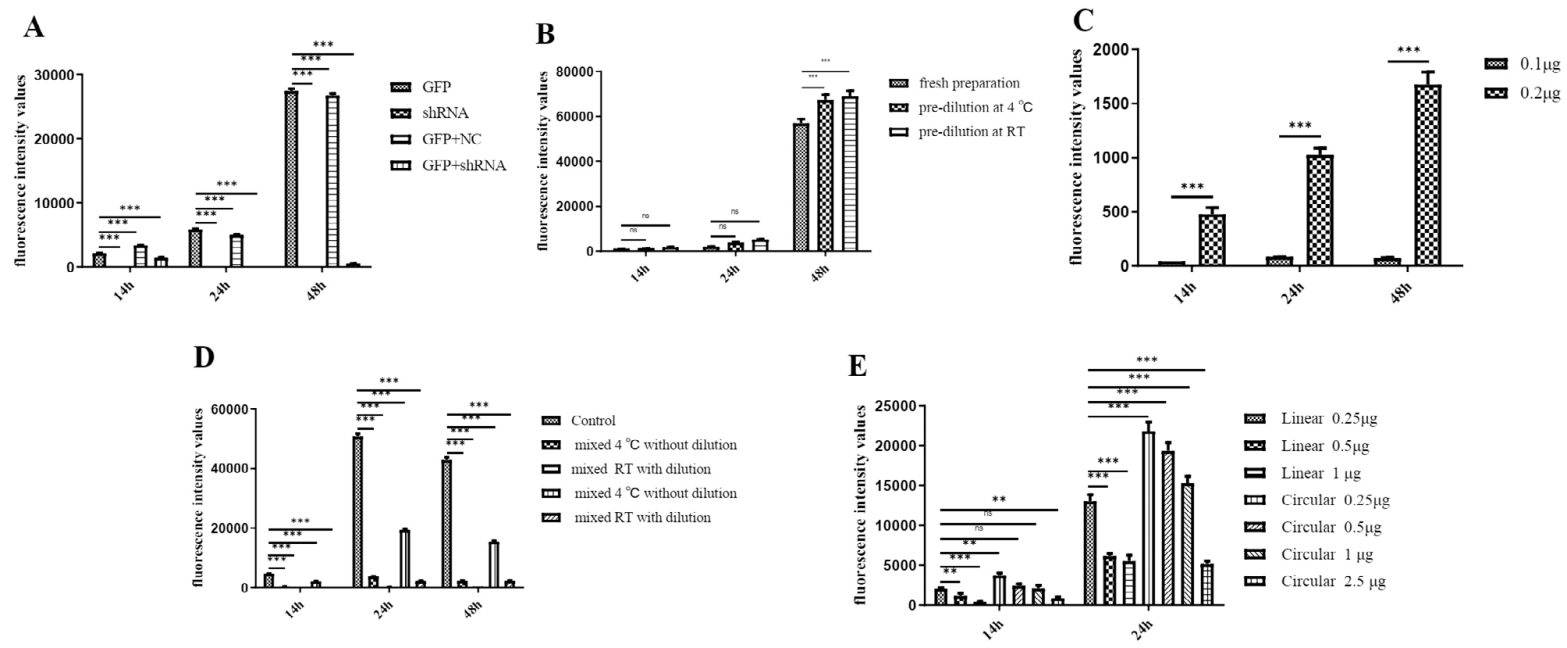
Fluorescence intensities of GFP-positive cells. (A) Fluorescence intensities of GFP-positive cells in the GFP, shRNA, GFP+NC, and GFP+shRNA groups at 14, 24, and 48 h (***p < 0.001). (B) Fluorescence intensities under the non-diluted, pre-diluted at 4 °C, and pre-diluted at room temperature conditions at 14, 24, and 48 h; ***p < 0.001; ns, not significant). (C) Student’s *t*-test for the comparison of 0.1 and 0.2 µg GFP at 14, 24, and 48 h; ***p < 0.001. (D) Fluorescence intensities in the control, mixed at 4 °C or room temperature without dilution, and mixed at 4 °C or room temperature with dilution groups (***p < 0.001). (E) Student’s *t*-test for comparison of the linear and circular small nucleic acids at 14 and 24 h. ns, not significant; **p < 0.01 and ***p < 0.001.

Compared with the control, transfection of recombinant plasmids encoding GFP-shRNA significantly reduced GFP expression to a nadir at 24 h (Figure 1). GFP expression was significantly lower in the GFP+shRNA group than in the other groups at 14, 24, and 48 h (***p < 0.001) (Figure 2).

### Evaluation of pre-diluted transfection reagent

**Figure 3 -:**
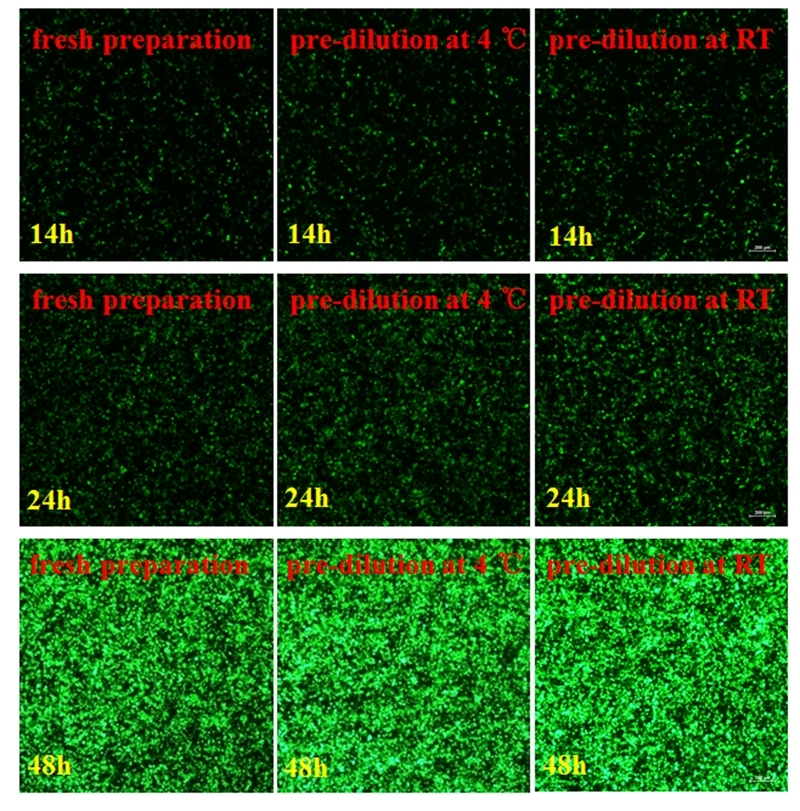
Among the non-diluted, pre-diluted at 4 °C, and pre-diluted at room temperature conditions, the transfection reagent diluted at room temperature showed the greatest transfection efficiency at 14, 24, and 48 h.

The transfection reagent was prepared under the following three conditions: non-diluted, pre-diluted at 4 °C, and pre-diluted at RT. A recombinant plasmid carrying GFP-shRNA was incubated with the transfection reagent, and transfection efficiency was analyzed after 14, 24, and 48 h. The transfection reagent pre diluted at RT showed the greatest transfection efficiency (Figure 3). The transfection reagent is diluted in advance, which has no effect on transfection (Figure 2). Also, storage at RT post dilution simplified the application process.

### Effect of plasmid concentration on transfection efficiency

**Figure 4 -:**
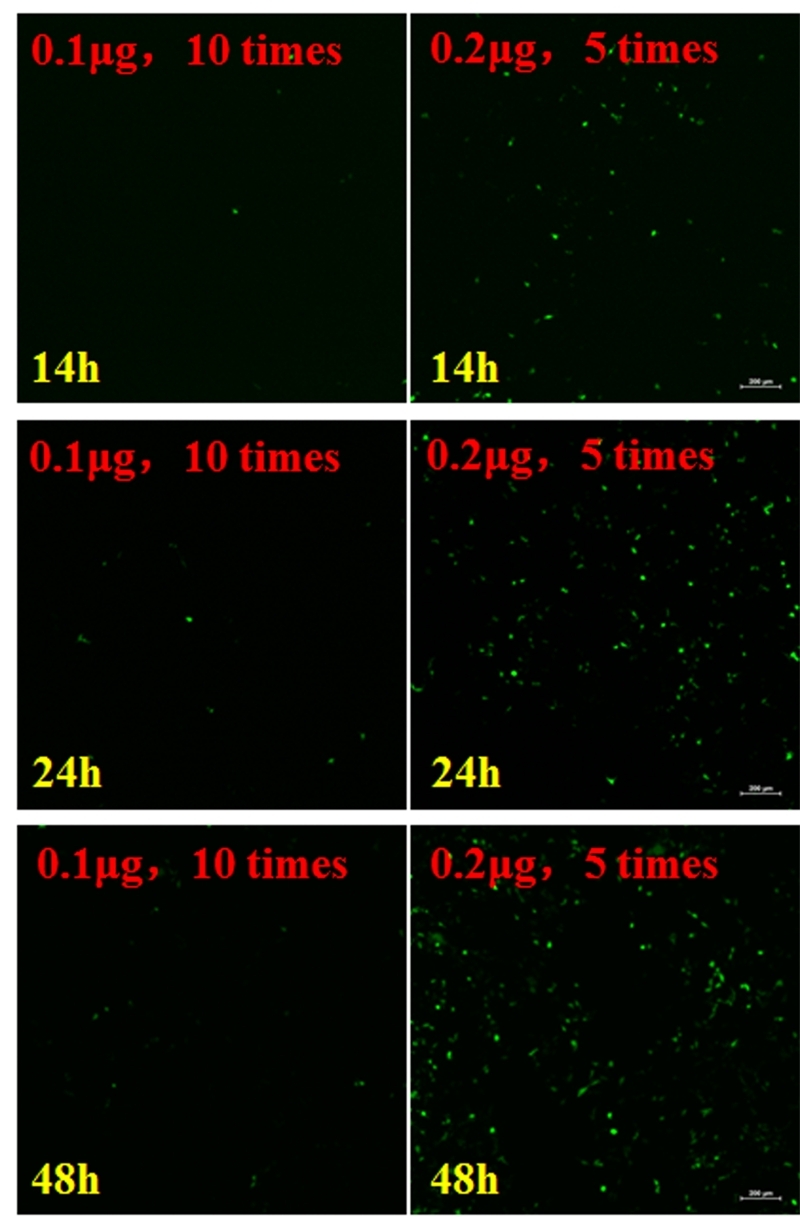
There were few GFP-positive cells after transfection of the GFP plasmid at 10 (0.1 g) - and 5 (0.2 g) - fold dilutions at 14, 24, and 48 h.

The GFP plasmid was diluted 10 (0.1 μg) - and 5 (0.2 μg) - fold and transfected into 293T cells. As shown in Figure 2, there were few GFP-positive cells, and the 10-fold diluted GFP resulted in the highest transfection efficiency. The 10-fold dilution of GFP plasmid reduced the number of GFP-positive cells, indicating impaired transfection (***p < 0.001) (Figures 2 and 4).

**Figure 5 -:**
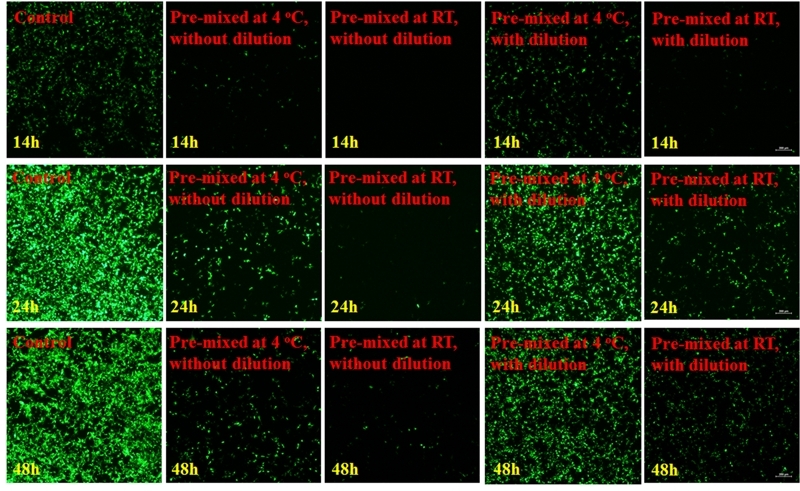
GFP was used as the simulant. The premixed solution was prepared, and half of the solution volume was diluted with PBS. Next, one quarter volumes of the diluted and non-diluted solutions were stored at 4 °C and another quarter volume at room temperature. After storage, the cells were transfected and analyzed after 14, 24, and 48 h.

Non-diluted small nucleic acids premixed at RT showed optimal transfection efficiency after 24 h (Figure 5). After premixing of the small nucleic acids, the system was unstable and failed at RT. For storage at 4 °C, small nucleic acids must first be mixed and diluted.

### Effects of linear and circular small nucleic acids

**Figure 6 -:**
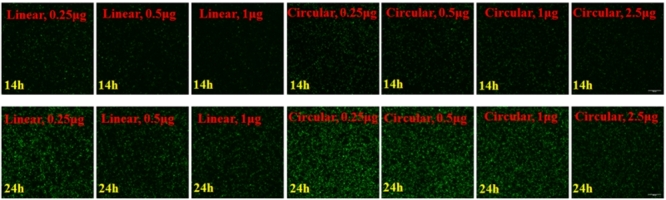
Using GFP as the target, linear and circular small nucleic acids were used for interference, and different gradients were used to compare the effects under the same mass and the same mole number. Transfection efficiency was analyzed at 14 and 24 h.

**Figure 7 -:**
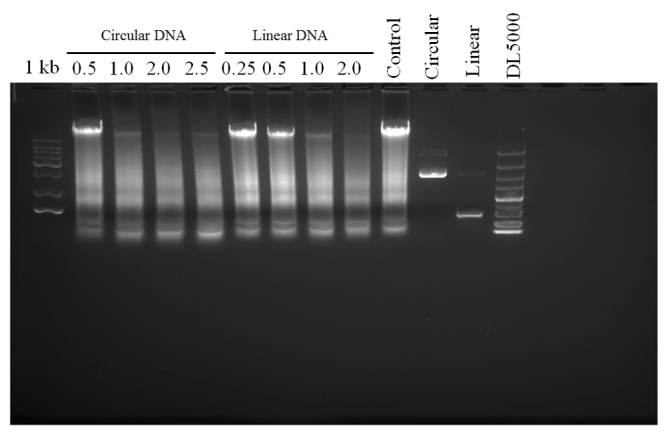
Genomic DNA was extracted, and genome insertion events were detected at 14 and 24 h.

Linear small nucleic acids showed greater transfection efficiency compared with circular small nucleic acids (Figure 2 and 6, ***p < 0.001), and the circular recombinant plasmid had better effect with more moles than linear. Also, linear small nucleic acids at 2 μg enabled complete genome insertion of the GFP gene, whereas circular small nucleic acids at 2.5 μg impaired expression of the GFP gene (Figure 7).

## Discussion

Cell transfection using cultured cells enables investigation of gene function. Recombinant plasmids carrying shRNA suppress gene expression via RNAi (Yu et al., 2013; Wu et al., 2016). The introduction of recombinant plasmids in cultured cells is key for cell transfection. Liposome-mediated transfection is a convenient method of transfecting plasmid DNA or RNA into eukaryotic cells, and it is associated with high transfection efficiency and repeatability and low cytotoxicity (Huang et al., 2015; Eloy et al., 2018). However, the transfection procedures differ according to cell type and lipid reagent (Dean and Gasiorowski, 2011; Ghanbari Safari and Hosseinkhani, 2013). In this study, we optimized the conditions of cell transfection mediated by EZ Trans Cell Reagent, a novel cationic polymer with low toxicity. The positively charged cationic polymers form positively charged complexes with negatively charged phosphate groups in nucleic acids, interact with negatively charged proteoglycans on the cell surface, and enter the cell by endocytosis.

A variety of lipid reagents have been developed for cell transfection, such as cationic lipids, cationic polymers, and nanoparticles, and significantly enhance transfection efficiency (Patel and Muthuswamy, 2012). Cationic lipids have a phospholipid bilayer structure, containing a positively charged head group and one or two hydrocarbon tails (Xia et al., 2016). Based on electrostatic interactions, cationic lipids are endocytosed (Ozpolat et al., 2014; Berardo et al., 2019). In this study, cell transfection was mediated by EZ Trans Cell Reagent. The transfection reagent pre-diluted at RT and the transfection reagent incubated with 0.1 μg GFP resulted in high transfection efficiencies.

Transfection time is pivotal for transfection efficiency. Transfection efficiency peaks at 24 or 48 h (Bottger et al., 2015) depending on the cell type, target gene transcription and translation levels, and the transfection reagent and its level of cytotoxicity. Compared with linear, the transfection effect is better with more moles and the dose effect will cause cytotoxicity. The high cell viability was probably attributed to the zeta potential and particle size of the conjugate (Alkan et al., 2020) and greater stability (O’Doherty et al., 2020).

For knockdown of specific genes in cultured cells, the shRNA sequence must be designed properly. In this study, introduction of shRNA and GFP significantly decreased GFP expression. Moreover, non-diluted small nucleic acids premixed at RT were superior to diluted small nucleic acids. In addition, linear small nucleic acids were superior to circular small nucleic acids, exhibiting a high rate of genome integration.

In conclusion, we optimized the conditions for cationic polymer-mediated transfection. Pre-diluted transfection reagent at RT, ≤ 0.1 μg plasmid, and non-diluted small nucleic acids premixed at RT yielded the highest transfection efficiency, which peaked at 24 h. In addition, linear small nucleic acids were superior to circular small nucleic acids and showed a high rate of genome integration.
